# Supplementary material for: Community perspectives regarding brain-computer interfaces: A cross-sectional study of community-dwelling adults in the UK
Source: PLOS Digit Health. 2025 Feb 5;4(2):e0000524. doi: 10.1371/journal.pdig.0000524 (PMC11798465; doi:10.1371/journal.pdig.0000524)
Supplement: S1 File — (DOCX) [file pdig.0000524.s001.docx]

Future Brain Study

Start of Block: Introduction & Consent

Q1
**Imperial College London Department of Primary Care & Public Health** is conducting a study to investigate public knowledge, attitudes & practices regarding Brain-Computer Interfaces (BCIs).
 
BCIs are devices that link the brain's electrical activity directly to computers or robotic limbs. Some BCIs can "translate" cerebral information into action, movement or communication. Others work the other way around & feedback external information (such as sounds) to the user's brain. BCIs can be used in medical research but also in entertainment, education or work to enhance individual capacities & abilities.
 We want to know how members of the community feel about BCIs generally & how BCIs could & should be used in the future, for whom & for what purposes.

 For more information, please refer to the Participant Information Sheet. Thank you for participating in this short (<10 min) survey.

Q2 Please confirm you agree with the below statements:
 I confirm that I have read & understand the participant information sheet version 0.2 dated 22/11/2023 for the above study & have had the opportunity to ask questions which have been answered fully I understand that my participation is voluntary & I am free to withdraw at any time, without giving any reason & without my legal rights nor healthcare being affected
I give consent for information collected about me to be used to support other research by an academic institution or commercial company in the future, including those outside of the United Kingdom (which Imperial has ensured will keep this information secure)
I understand that data collected from me are a gift donated to Imperial College & that I will not personally benefit financially if this research leads to an invention and/or the successful development of a new test, medication treatment, product or service
I consent to take part in this study.

- Yes (1)
- No (2)

Skip To: End of Survey If Please confirm you agree with the below statements: I confirm that I have read & understand the p... = No

End of Block: Introduction & Consent

Start of Block: Knowledge & personal experience

Q3 Have you ever used a Brain Computer Interface (BCI)?

- Yes, I am currently using one (1)
- Yes, but I no longer use it (2)
- No, never (3)

Display This Question:

If Have you ever used a Brain Computer Interface (BCI)? != No, never

Q4 What type of BCI technology have you been using? (Please select all that apply)

- Electroencephalography (EEG) (1)
- Magnetoencephalography (MEG) (2)
- Functional magnetic resonance imaging (fMRI) (4)
- Electrocorticography (ECoG), a type of intracranial electroencephalography (iEEG) (5)
- Microelectrode arrays (MEAs) (3)
- I'm not sure (6)

Display This Question:

If Have you ever used a Brain Computer Interface (BCI)? != No, never

Q5 For what purpose have you been using a BCI? (Please select all that apply)

- For medical purposes (please specify) (1) __________________________________________________
- For non-medical purposes (please specify) (2) __________________________________________________

Q6 How much do you know about BCIs? Please select the option that best describes you.

- Nothing, I had never heard of BCIs before (1)
- I don't know much but I have already heard of BCIs (4)
- I know a lot about BCIs (5)

Skip To: Q10 If How much do you know about BCIs? Please select the option that best describes you. = Nothing, I had never heard of BCIs before

Display This Question:

If How much do you know about BCIs? Please select the option that best describes you. != Nothing, I had never heard of BCIs before

Q7 Which of the following BCI devices have you heard about? (You can select more than one)

- Hearing aid with brain implant component (1)
- Neuralink (Owned by Elon Musk) (2)
- Synchron (Stentrode) (3)
- Others (please specify) (4) __________________________________________________
- None of the above (5)

Display This Question:

If How much do you know about BCIs? Please select the option that best describes you. != Nothing, I had never heard of BCIs before

Q8 Which of the following BCI technologies have you heard about? (Please select all that apply)

- Electroencephalography (EEG) (1)
- Magnetoencephalography (MEG) (2)
- Functional magnetic resonance imaging (fMRI) (4)
- Electrocorticography (ECoG), a type of intracranial electroencephalography (iEEG) (5)
- Microelectrode arrays (MEAs) (3)
- None of the above (6)

Display This Question:

If How much do you know about BCIs? Please select the option that best describes you. != Nothing, I had never heard of BCIs before

Q9 Where have you heard or read about BCIs?

- In the news (on TV, radio, online or printed newspaper) (1)
- Through advertisement (6)
- In the scientific literature (7)
- On online forums (2)
- From friends & family (3)
- From colleagues at work (4)
- From my GP, nurse, or another healthcare professional (5)
- Other (please specify) (8) __________________________________________________

Q10 In which of the following fields do you think BCIs **are already being used**? (Please select all that apply)

- Health care & assistive technology (e.g.: to support patients with paralysis or for stroke rehabilitation) (1)
- Education & learning (e.g.: to address learning disabilities) (2)
- Entertainment & recreational use (e.g.: for gaming or sports) (3)
- Workplace (e.g.: to track & improve productivity) (4)
- Marketing & commerce (e.g.: for targeted advertisement & personalised needs assessments) (5)
- Military, police & security use (e.g.: lie detection) (6)
- None of the above (8)
- Other (please specify) (7) __________________________________________________

Q11 In which of the following fields do you think BCIs **are currently being designed & tested (still at the experimental stage & not yet being used)**? (Please select all that apply)

- Health care & assistive technology (e.g.: to support patients with paralysis, stroke) (1)
- Education & learning (e.g.: to address learning disabilities) (2)
- Entertainment & recreational use (e.g.: gaming, sports) (3)
- Workplace (e.g.: to track & improve productivity) (4)
- Marketing & commerce (e.g.: for targeted advertisements) (5)
- Military, police & security use (e.g.: lie detection) (6)
- None of the above (7)
- Other (please specify) (8) __________________________________________________

End of Block: Knowledge & personal experience

Start of Block: Attitudes & perceptions

Q12 In which of the following fields do you think BCIs **should** be designed, tested & used? (Please select all that apply)

- Health care & assistive technology (e.g.: to support patients with paralysis, stroke) (1)
- Education & learning (e.g.: to address learning disabilities) (2)
- Entertainment & recreational use (e.g.: gaming, sports) (3)
- Workplace (e.g.: to track & improve productivity or communication) (4)
- Marketing & commerce (e.g.: for targeted advertisements) (5)
- Military, police & security use (e.g.: lie detection) (6)
- None of the above (7)
- Other (please specify) (8) __________________________________________________

Q13 **Non-invasive BCIs** refer to electrodes placed on the head using a headband or helmet which are easily removable & do not require any surgical procedure. On the other hand, **invasive BCIs** are electrodes placed directly in the brain, they are permanent, more reliable & accurate but they also carry more risk because of the need for surgery to implant them.

 For each of the following, what type of BCI would you personally consider using? (you can select both types where appropriate)

|  | Non-invasive BCI (1) | Invasive BCI (2) | I would not consider using any BCI (3) |
| --- | --- | --- | --- |
| Complete paralysis (1) |  |  |  |
| Partial paralysis (10) |  |  |  |
| Weakness in my arms or legs (2) |  |  |  |
| Stroke rehabilitation (15) |  |  |  |
| Speech impediment (4) |  |  |  |
| Visual impairment (9) |  |  |  |
| Lack of control of my bladder (7) |  |  |  |
| Lack of control of my bowels (8) |  |  |  |
| Parkinson's disease (13) |  |  |  |
| Attention-deficit hyperactivity disorder (ADHD) (14) |  |  |  |
| Dementia (3) |  |  |  |
| Depression (5) |  |  |  |
| Anxiety (6) |  |  |  |
| To enhance my physical abilities (strength, speed) (17) |  |  |  |
| To enhance my cognitive abilities (memory, attention, etc) (18) |  |  |  |

Q14 Would you consider getting a BCI for any other reason not listed above?

- No (4)
- Yes (please specify) (5) __________________________________________________

Q15 What would prevent you from getting an invasive BCI (requiring a surgical procedure)? Please select all that apply

- High cost of buying & maintaining the device (1)
- Stigma of having a medical device implanted (3)
- Complexity & risk of the surgical procedure to IMPLANT the BCI (4)
- Complexity & risk of the surgical procedure to REMOVE the BCI (19)
- Postoperative care (including attending further appointments) (14)
- Risks in case the BCI stops working or malfunctions (5)
- Limited effectiveness of the BCI (21)
- Concern the BCI may alter my personality (7)
- Concern the BCI may alter my mood (8)
- Lack of evidence & historical perspective regarding BCIs (9)
- Enabling further access to personal data (15)
- Risk of hacking of the device (16)
- General distrust in BCIs & the companies selling them (10)
- Aesthetics (it may not look good) (2)
- Lack of support or agreement from my family & friends (24)
- Lack of support or agreement from my religious community (25)
- Other (please specify) (17) __________________________________________________

Carry Forward Selected Choices from "What would prevent you from getting an invasive BCI (requiring a surgical procedure)? Please select all that apply"

| 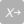 |
| --- |

Q16 Using the sliding scale below, where 1=least important and 5=most important, please rate how important the following factors would be in influencing your decision to get an invasive BCI:

|  | Least important | Most important |
| --- | --- | --- |

|  | 1 | 2 | 3 | 4 | 5 |
| --- | --- | --- | --- | --- | --- |

| Equitable access of BCIs () | 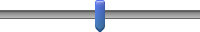 |
| --- | --- |
| High cost of buying & maintaining the device () | 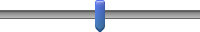 |
| Stigma of having a medical device implanted () | 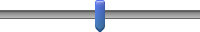 |
| Complexity & risk of the surgical procedure to IMPLANT the BCI () | 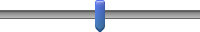 |
| Complexity & risk of the surgical procedure to REMOVE the BCI () | 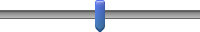 |
| Postoperative care (including attending further appointments) () | 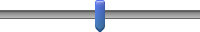 |
| Risks in case the BCI stops working or malfunctions () | 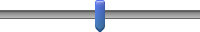 |
| Limited effectiveness of the BCI () | 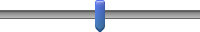 |
| Concern the BCI may alter my personality () | 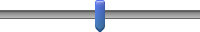 |
| Concern the BCI may alter my mood () | 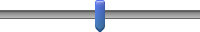 |
| Lack of evidence & historical perspective regarding BCIs () | 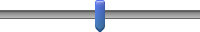 |
| Enabling further access to personal data () | 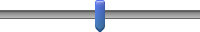 |
| Risk of hacking of the device () | 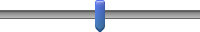 |
| General distrust in BCIs & the companies selling them () | 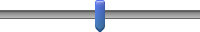 |
| Aesthetics (it may not look good) () | 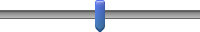 |
| Lack of support or agreement from my family & friends () | 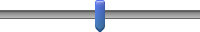 |
| Lack of support or agreement from my religious community () | 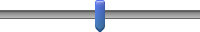 |
| Other (please specify) () | 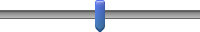 |

Q17 How long do you think it will be before BCIs become the "new normal" in the UK, in the sense that not all but the great majority of the UK population will be using them (similar to smartphones nowadays)?

- Less than a year (1)
- 2 to 5 years (2)
- 6 to 10 years (3)
- More than 10 years (4)
- BCIs will never be used so broadly (5)

Q18 To what extent do you agree with the following statements regarding BCIs in general (both invasive and non-invasive)?

|  | Strongly disagree (1) | Disagree (2) | Neither agree nor disagree (3) | Agree (4) | Strongly agree (5) |
| --- | --- | --- | --- | --- | --- |
| I'm worried about the effect of BCIs being widely available to the public (3) |  |  |  |  |  |
| I'm excited for the potential that BCIs can bring for society (4) |  |  |  |  |  |
| People with BCIs will be more productive in their work (5) |  |  |  |  |  |
| People with BCIs will feel superior to those without (6) |  |  |  |  |  |
| BCIs will lead to an increase in inequalities (7) |  |  |  |  |  |
| I'm worried BCIs may be implanted without proper consent (23) |  |  |  |  |  |
| BCIs will increase stigmatisation & pathologisation of people with disability (24) |  |  |  |  |  |
| Use of invasive BCIs in healthy patients is morally wrong (9) |  |  |  |  |  |
| Invasive BCIs should be reserved to people with physical and/or cognitive disabilities (16) |  |  |  |  |  |
| I support strict regulation in the development & use of BCIs even if it means technological progress is slowed (10) |  |  |  |  |  |
| BCIs for medical purposes should be reimbursed by the NHS or insurance companies (13) |  |  |  |  |  |
| BCIs for healthy people should be reimbursed by the government or insurance companies (14) |  |  |  |  |  |
| BCIs should not be accessible to children (under 18 years old) (21) |  |  |  |  |  |

End of Block: Attitudes & perceptions

Start of Block: Demographic

Q19 What is your age (in years)?

▼ 18 (187) ... 107 (364)

Q20 What is your gender?

- Male (1)
- Female (2)
- Other (please feel free to specify) (4) __________________________________________________
- Prefer not to say (5)

Q21 What best describes your ethnic group or background?

- Asian, or Asian British (4)
- Black, Black British, Caribbean or African (5)
- Mixed or Multiple ethnic groups (2)
- White (1)
- Other ethnic groups (please specify) (6) __________________________________________________
- Prefer not to say (7)

| Page Break |  |
| --- | --- |

Q22 What is your religion?

- No religion (1)
- Buddhism (3)
- Catholicism (11)
- Hinduism (4)
- Judaism (5)
- Islam (6)
- Protestantism (10)
- Sikhism (7)
- Other religion (please specify) (8) __________________________________________________
- Prefer not to say (9)

Q23 How important is religion in your life?

- Very important (1)
- Important (2)
- Neither important nor unimportant (3)
- Unimportant (4)
- Very unimportant (5)

Q24 Where do you live?

- England (1)
- Northern Ireland (2)
- Scotland (3)
- Wales (4)
- Outside of UK (5)

| Page Break |  |
| --- | --- |

Q25 Do you consider yourself to have a disability?

- No (1)
- Yes & with paralysis (please feel free to specify) (4) __________________________________________________
- Yes, but without paralysis (please feel free to specify) (2) __________________________________________________
- Prefer not to say (3)

Q26 Do you have a relative or friend with disability?

- No (1)
- Yes & with paralysis (please feel free to specify) (3) __________________________________________________
- Yes but without paralysis (please feel free to specify) (2) __________________________________________________
- Prefer not to say (4)

Q27 What is your highest level of education?

- Primary school (1)
- Secondary school up to 16 years (2)
- Higher or secondary or further education (A-levels, BTEC, etc.) (4)
- College or university degree (5)
- Prefer not to say (3)

| Page Break |  |
| --- | --- |

Q28 What is your current employment status?

- Employed full-time (1)
- Employed part-time (2)
- Unemployed (3)
- Retired (4)
- Student (6)
- Prefer not to say (5)

End of Block: Demographic

Start of Block: Consent to contact

Q29 Thank you for taking the time to answer our questionnaire. Researchers from Imperial College London are looking to carry individual interviews and focus groups with 10 to 15 participants (via telephone, Skype or Microsoft Teams) to learn more about your perspectives on BCIs. Interviews will last 30-45 minutes approximately while focus groups will last around 1-hour. The format (individual interview or focus group) in which you will take part will depend on your personal preference and availability. Please provide your name & contact details if this interests you so we may contact you to fix a suitable time for you to take part in an individual interview or focus group. If you are unsure or have any questions, we will be happy to answer you.

- Name: (1) __________________________________________________
- Email address: (2) __________________________________________________

End of Block: Consent to contact
