# Supplementary material for: Community perspectives regarding brain-computer interfaces: A cross-sectional study of community-dwelling adults in the UK
Source: PLOS Digit Health. 2025 Feb 5;4(2):e0000524. doi: 10.1371/journal.pdig.0000524 (PMC11798465; doi:10.1371/journal.pdig.0000524)
Supplement: S1 Table — (DOCX) [file pdig.0000524.s004.docx]

## S1 TABLE

**Respondent characteristics (N=806)**

| **Age** | N | (%) |
| --- | --- | --- |
| 18-25 | 100 | (12.4) |
| 26-35 | 211 | (26.2) |
| 36-45 | 213 | (26.4) |
| 46-55 | 133 | (16.5) |
| 56-65 | 99 | (12.3) |
| 66 and older | 50 | (6.2) |
| **Gender** | | |
| Female | 416 | (51.6) |
| Male | 386 | (47.9) |
| Non-binary | 1 | (0.1) |
| Prefer not to say | 3 | (0.4) |
| **Ethnicity** | | |
| White | 690 | (85.6) |
| Mixed/Multiple ethnic groups | 11 | (1.4) |
| Asian/Asian British | 58 | (7.2) |
| British Black/African/Caribbean | 37 | (4.6) |
| Other | 6 | (0.7) |
| Prefer not to say | 4 | (0.5) |
| **Religion** |  |  |
| Christian | 191 | (23.7) |
| Buddhism | 7 | (0.9) |
| Hinduism | 9 | (1.1) |
| Islam | 26 | (3.2) |
| Sikhism | 2 | (0.3) |
| No religion | 538 | (66.7) |
| Other religion | 14 | (1.7) |
| Prefer not to say | 19 | (2.4) |
| **Importance of religion in life (religiosity)** |  |  |
| Unimportant | 556 | (69.0) |
| Neither important nor unimportant | 117 | (14.5) |
| Important | 133 | (16.5) |
| **Residence** |  |  |
| England | 684 | (84.8) |
| Northern Ireland | 16 | (2.0) |
| Scotland | 70 | (8.7) |
| Wales | 36 | (4.5) |
| **Disability** |  |  |
| Yes & with paralysis | 3 | (0.4) |
| Yes, but without paralysis | 87 | (10.8) |
| No | 701 | (87.0) |
| Prefer not to say | 15 | (1.8) |
| **Relative or friend with a disability** |  |  |
| Yes & with paralysis | 35 | (4.3) |
| Yes, but without paralysis | 188 | (23.3) |
| No | 556 | (69.0) |
| Prefer not to say | 27 | (3.4) |
| **Education** |  |  |
| Primary school | 2 | (0.3) |
| Secondary school up to 16 years | 81 | (10.0) |
| Higher or secondary or further education (A-levels, BTEC, etc.) | 182 | (22.6) |
| College or university degree | 536 | (66.5) |
| Prefer not to say | 5 | (0.6) |
| **Employment status** |  |  |
| Employed full-time | 448 | (55.6) |
| Employed part-time | 149 | (18.5) |
| Retired | 66 | (8.2) |
| Student | 42 | (5.2) |
| Unemployed | 85 | (10.5) |
| Prefer not to say | 16 | (2.0) |
