# Supplementary material for: Community perspectives regarding brain-computer interfaces: A cross-sectional study of community-dwelling adults in the UK
Source: PLOS Digit Health. 2025 Feb 5;4(2):e0000524. doi: 10.1371/journal.pdig.0000524 (PMC11798465; doi:10.1371/journal.pdig.0000524)
Supplement: Attachment — Submitted filename: Response to Reviewers.docx [file pdig.0000524.s005.docx]

September 2024

Avinash Singh, PhD

Academic Editor, *PLOS Digital Health*

Dear Editor,

I appreciate the chance to revise and resubmit our manuscript titled “**Community Perspectives Regarding Brain-Computer Interfaces: A Cross-Sectional Study of Community-Dwelling Adults in the UK**.” We are thrilled to have received such encouraging feedback and wish to extend our sincere thanks to you and the reviewers for your thoughtful insights and constructive recommendations.

We fully acknowledge the reviewers’ comments and have addressed each point raised. Enclosed, you will find our detailed responses, along with a Revised Manuscript that highlights the changes made.

We believe that these valuable suggestions have significantly improved the manuscript, and we hope it is now ready for publication in your prestigious journal.

Thank you once more for your support.

On behalf of authors,

Austen El Osta

Corresponding Author

**RESPONSES TO REVIEWER COMMENTS**

| **Reviewer 1** | | |
| --- | --- | --- |
|  | **Comment** | **Response** |
|  | The paper presents a comprehensive survey on public perspectives regarding Brain-Computer Interfaces (BCIs) among community-dwelling adults in the UK. The paper is well-written and easy to understand. | Thank you for your constructive feedback. |
|  | Discussion on Non-Response Bias: The paper reports a high response rate, but there is little discussion on the potential for non-response bias. | Thank you for highlighting this. We have calculated response rate and we acknowledged that non-response bias as a limitation. We also considered that whilst our study had a high response rate, nonresponse bias may still pose a concern as the characteristics and opinions of who did not participate could differ from those who did, potentially skewing our study findings. We updated the limitation section in the revised manuscript to reflect this. |
|  | Validity of the Results: Did the authors ensure that the results of a survey accurately reflect the perspectives of the targeted population in some ways? | Thank you for your insightful question regarding the validity of our survey results. To ensure that our findings accurately reflect the perspectives of the targeted population, we highlighted in the methods section that we conducted a pilot study to refine our survey questions and methodology, ensuring they effectively capture the relevant insights. Further, we reiterated that the end of the survey, we included a question inviting respondents to express their interest in participating in personal interviews. We are currently in the process of collecting interview data which will deepen our understanding of the community’s perspectives. The contextual findings will complement the quantitative data presented in this (quantitative analysis) paper. We plan to publish these results in PLOS (earmarked for submission in late 24/early 25), to provide a comprehensive view of the community’s insights and enriching the existing knowledge in this field. |
|  | Figures and Graphical Representations: Adding figures to visually represent the results, such as bar graphs of demographic breakdowns or pie charts showing levels of support for different applications of BCIs, would make the data more accessible and easier to interpret at a glance. | Thank you for highlighting this. We have included two figures to the revised manuscript. The first figure is a bar chart comparing public perceptions of BCIs applications in different fields across three categories: current use, experimental stag, and desired future use. The second figure is a funnel chart displays the perceived barriers to adopting BCIs, ranked by percentage of concern among respondents. |

| **Reviewer 2** | | |
| --- | --- | --- |
|  | **Comment** | **Response** |
|  | The title is clear but why using question format. | Thank you for highlighting this. We adjusted the title of the paper to: "Community Perspectives on Brain-Computer Interfaces: A Cross-Sectional Study of Community-Dwelling Adults in the UK." |
|  | The abstract is concise and provides a clear summary of the study’s objectives, methods, and key findings. However, including specific numerical results and a brief mention of the main conclusions is needed. | Thank you for your suggestion. We revised the abstract to include specific numerical results and a brief mention of the main conclusions. The updated abstract can be found in the revised manuscript. |
|  | The study design is robust. However, the inclusion of a pilot test for the survey instrument should be mentioned to validate the questionnaire’s reliability and comprehensibility. | Thank you for this observation. We updated the manuscript to mention that a pilot test was conducted to ensure the reliability and comprehensibility of the survey instrument. The questionnaire was reviewed by two academic expert researchers to determine its suitability, consistency and validity. As is the procedure for all our eSuvey based studies, the questionnaire was piloted with 25 eligible participants, who were subsequently removed from the study. Feedback from experts was utilized to revise the questionnaire to eliminate any ambiguity. The data collected during this initial pilot study was not included in the final analysis. |
|  | The results section is comprehensive. However, the narrative could benefit from a more explicit linkage of the results to the study’s aims and research questions. | We appreciate this feedback. We have revised the results section to more explicitly link the findings to the study’s aims and research questions. "The results section now explicitly links the findings to the study’s aims and research questions. For example, the strong interest in medical applications of BCIs directly addresses the research question regarding public attitudes toward different BCI applications." |
|  | The discussion needs a deeper exploration of the reasons behind the public’s mixed perceptions and the potential impact of demographic variables on attitudes towards BCIs. | Thank you for this valuable suggestion. We expanded the discussion to include a deeper exploration of the reasons behind the public’s mixed perceptions and analysed the potential impact of demographic variables on attitudes towards BCIs. |
|  | The conclusion is missing specific recommendations for policymakers, researchers, and practitioners about its practical relevance. | Thank you for pointing this out. We revised the conclusion to include specific recommendations for policymakers, researchers and practitioners, emphasizing the practical relevance of the study’s findings. |
|  | The sampling method is not clearly described. It is unclear whether random, convenience, or purposive sampling was used. | We clarified the sampling method in the manuscript, specifying that a convenience sampling method was used to recruit participants via various online platforms and network. The sampling method employed for this study was convenience sampling, allowing us to gather data from individuals who were readily accessible and willing to participate. |
|  | The sample size of 806 is mentioned but there is no justification for this number. A power calculation to determine the appropriate sample size is missing. | Thank you for highlighting this. We calculated the sample size in the revised manuscript using Raosoft online software with a margin of error= 5%, confidence interval= 95%, and response distribution= 50%; thus, the recommended sample size was 385 participants. Our study exceeded this sample nearly threefold. |
|  | There is no information on the validity and reliability of the questionnaire used. | We acknowledge the importance of this aspect. The validity and reliability of the questionnaire were ensured through a pilot test, which has now been mentioned in the revised manuscript.  The questionnaire was reviewed by two academic expert researchers to determine its suitability, consistency, and validity. As is the procedure for all our survey based studies, the questionnaire was piloted with 25 eligible participants, who were subsequently removed from the study. Feedback from experts was utilized to revise the questionnaire to eliminate any ambiguity. The data collected during this initial pilot study was not included in the final analysis. |
|  | The method of administration (online, face-to-face, etc.) is not specified. | The method of administration was entirely online using the Qualtrics platform, as described in the methodology section of the manuscript.  The survey was administered entirely online using the Qualtrics platform. This method facilitated wide distribution and ensured data collection in a standardized format, accessible to a broad demographic. |
|  | The response rate is not reported, which is crucial for assessing the representativeness of the sample. | The response rate has now been reported in the revised manuscript. 806 out of 950 potential eligible participants who completed the survey, with a response rate of 84.84%. |
|  | Ethical approval and informed consent procedures are not mentioned, raising concerns about the ethical rigor of the study. | The study received a favourable opinion from the Imperial College London Research Ethics Committee, as mentioned in the revised manuscript (Ethics Approval #6887726). Participants provided consent to take part in the survey.  The study was approved by the Imperial College London Research Ethics Committee (ICREC# 6887726). Informed consent was obtained from all participants before they proceeded with the survey, ensuring ethical compliance throughout the study. |
|  | The results section is primarily descriptive with limited inferential statistics. The study does not seem to leverage advanced statistical analyses to draw more robust conclusions. | Thank you for your valuable feedback. We appreciate your insights regarding the results section and the use of inferential statistics. We would like to clarify that we conducted Chi-square test & a statistical hypothesis test to analyse the associations between various demographic factors and key perceptions regarding Brain-Computer Interfaces (BCIs). Specifically, we examined:   - The relationship between demographics and the belief that BCIs will lead to increased inequalities. - The association between demographics and support for strict regulations in the development and use of BCIs, even if it may hinder technological progress. - The links between demographics and the fields of healthcare and assistive technology where BCIs should be designed, tested, and utilized.   We believe that the Chi-square test is particularly suitable for our analyses as it allows for the examination of relationships between categorical variables, which is precisely the nature of the data we collected. Thank you again for your constructive feedback. |
|  | There is a lack of clarity in the presentation of demographic data and their implications on the study’s findings. | We revised the demographic data presentation to enhance clarity and provided a more detailed analysis of how these demographics impact the study’s findings. This is now detailed in the results and discussion sections. |
|  | Tables and figures are poorly integrated into the text and do not enhance the understanding of the results. | Thank you for this feedback. We revised the manuscript, text & flow to ensure that tables and figures are now better integrated to enhance readability and the understanding of the results. Each table and figure is now referenced and discussed in the context of the surrounding narrative, improving the flow and comprehension of the information presented |
|  | The reported percentages in the tables do not always add up to 100%, indicating potential errors in data presentation or analysis. | Thank you for pointing this out. We revised the reported percentages in the tables to make sure that percentages always add up to 100%. |
|  | The discussion is superficial and does not critically engage with the findings. | We revised the discussion to include a more critical engagement with the findings, addressing the implications and potential limitations of the study. |
|  | There is a lack of comparison with previous studies, which limits the study’s contribution to the existing body of knowledge. <https://doi.org/10.1371/journal.pone.0296884> <https://doi.org/10.1186/s12910-024-01062-8> | Thank you for pointing this out. We have compared our findings with previous studies, particularly those conducted on BCIs, and in similar populations. This comparison is detailed in the discussion section, where we highlight consistencies and differences between our results and those in the existing literature. We have included these references in the paper also. |
|  | Potential biases and limitations are either underexplored or ignored altogether. | We expanded the discussion on potential biases and limitations, including non-response bias, sampling bias and the limitations inherent to self-reported data. This is now detailed in the revised manuscript. |
|  | The implications of the findings for future research, policy, and practice are not adequately discussed. | We revised the conclusion and discussion sections to better address the implications of our findings for future research, policy, and practice, providing specific recommendations for each area. |
|  | The conclusion is weak and does not effectively summarize the main findings or their significance. | The conclusion has been strengthened to more effectively summarize the main findings and their significance, and to provide clear recommendations for future research and practice. |
|  | Recommendations for future research are vague and lack specificity. | We revised the recommendations for future research to be more specific, outlining clear paths for further investigation based on our findings. |
|  | The overall structure of the paper lacks coherence, making it difficult to follow the logical flow of arguments. | We reorganized the manuscript to improve coherence and ensure a logical flow of arguments from the introduction through to the conclusion. |
|  | Language and grammar issues are present throughout the manuscript, which detracts from the professionalism of the paper. | The manuscript has been thoroughly reviewed and edited to correct language and grammar issues, enhancing the professionalism and readability of the paper. |
|  | There is a noticeable absence of critical engagement with the literature and a lack of depth in the analysis. | We revised the manuscript to include more critical engagement with the relevant literature and have deepened the analysis to address this concern. |
|  | The study's contribution to the field is minimal due to its methodological flaws and superficial treatment of the topic. | The methodological concerns have been addressed, and the discussion has been expanded to provide a more thorough treatment of the topic. This revision enhances the study’s contribution to the field by providing a nuanced understanding of public perceptions of BCIs. |

| **Reviewer 3** | | |
| --- | --- | --- |
|  | **Comment** | **Response** |
|  | This is a very investing study and I was very happy to see that the authors directly engage with some of the concerns that the public might have about BCI and related applications. I think that the conclusions reached in the study are very interesting and show a direct engagement with these issues. I recommend publication and only have smaller suggestions. | Thank you for your feedback & constructive comments. |
|  | First, I wonder if the authors could say a bit more on the consequences of their findings for research and perhaps their research:  What should be done considering the reservations about the ethical, privacy and safety implications?  Could a path towards co-design or ethically-informed design be developed? | Thank you for this important suggestion. We expanded the discussion to address the consequences of our findings for future research, particularly in relation to ethical, privacy and safety implications. We have also included a discussion on the potential for co-design or ethically-informed design as a path forward. |
|  | Secondly and relatedly, the paper seems to imply that the identified issues are due to and will be resolved by more awareness and knowledge about the technology. I am not so sure about this, as some of the concerns seem to be aligned with how the technology works or at least could be developed. I wonder if the authors could say more about the idea of a gap between the potential of the technology and public awareness – to me it seems that the public was actually mostly aware of the potential of the technology and still concerned. | We appreciate this nuanced perspective. We revised the discussion to consider that public concerns may not merely be due to a lack of awareness or knowledge but could also be rooted in deeper issues related to the technology’s fundamental nature and development. This is reflected in the revised discussion section. |
